# Low-dose azathioprine is effective in maintaining remission among Chinese patients with Crohn’s disease

**DOI:** 10.1186/1479-5876-11-235

**Published:** 2013-09-27

**Authors:** Jianghong Wu, Yan Gao, Chuanhua Yang, Xueqing Yang, Xuhang Li, Shudong Xiao

**Affiliations:** 1 Division of Gastroenterology and Hepatology, Ren Ji Hospital, School of medicine, Shanghai Jiao Tong University, Shanghai Institute of Digestive Disease; Key Laboratory of Gastroenterology & Hepatology, Ministry of Health (Shanghai Jiao-Tong University), Shanghai, China; 2 Division of Gastroenterology and Hepatology, Tengzhou Central People’s Hospital, Tengzhou, China; 3 Division of 2008 undergraduate, Anhui Medical University, Hefei, China; 4 Department of Medicine GI Division, Johns Hopkins University School of Medicine, Baltimore, USA

**Keywords:** <1.0 mg/kg azathiopurine, Maintain remission, Crohn’s disease, Chinese patients

## Abstract

**Background:**

Azathiopurine (AZA) is efficacious for maintenance remission of Crohn’s disease (CD) at the standard dose of 2.0-2.5 mg/kg for Caucasian. It has been reported that the lower dose (1.0-2.0 mg/kg) in some Asian countries was as effective as the standard dose. In the present study we analyzed the efficacy of <1.0 mg/kg AZA in maintaining remission for Chinese patients.

**Methods:**

The clinical data of all CD patients were reviewed from 1993 to December 2012. The patients who initiated AZA treatment and were followed for ≥ 2 years with complete medical data were included. We divided the patients into two groups according to their initial dose: <1.0 mg/kg group and 1.0-2.0 mg/kg group.

**Results:**

Among 77 patients, 39 (50.6%) started treatment with <1.0 mg/kg AZA and 38 (49.4%) with 1.0-2.0 mg/kg. The mean dose of <1.0 mg/kg group remained under 1.0 mg/kg at 6, 12 and 24 months, even if the doses were adjusted according to efficacy and tolerance. The remission rate in patients of <1.0 mg/kg group was significantly higher than that in those of 1.0-2.0 mg/kg group (*P* = 0.025). A dose of <1.0 mg/kg AZA was more commonly associated with male gender, older age, heavier body weight and L1 location. Adverse events were observed in 21 of 77 patients (27.3%) and no significant difference in occurrence of adverse events or leucopenia between two groups.

**Conclusions:**

<1.0 mg/kg AZA was effective as 1.0-2.0 mg/kg in maintaining remission among Chinese patients with CD.

## Background

Crohn’s disease (CD) is an idiopathic chronic gastrointestinal inflammation of unknown etiology. Currently, the goal of treatment is to control disease activity by inducing and maintaining remission. In order to minimize occurrence of the associated complications or the need for surgery, patients have to take long-term medicines to control activity of disease and prevent relapse. For the maintenance of remission, immunomodulators (such as thiopurines and methotrexate) and anti-tumor necrosis factor-alpha monoclonal antibody have proven to be highly efficient [1,2]. They not only decrease CD clinical activity but also heal the mucosa of ulcers and erosions [3,4], reduce the need for corticosteroids, and improve the patient’s quality of life. In Asian countries, particularly in developing countries such as China, anti-tumor necrosis factor-alpha monoclonal antibody therapy is very expensive, and most families can’t afford it, because their health insurance doesn’t cover this therapy [5]. Thus, cheaper and highly effective immunomodulators, particularly azathiopurine (AZA), are widely accepted as the first-line treatment by both patients and gastroenterologists. Although severe side effects may occur [6,7], AZA has been widely used globally for CD therapy.

In European patients, 2.0-2.5 mg/kg of AZA has been shown to be effective against CD [8], and 1.0 mg/kg of AZA has been confirmed to have no benefit [9]. However, in Japan, low doses (50–100 mg daily) of AZA are recommended because Japanese patients are more susceptible to dose-dependent adverse events [10]. Although a higher dose of AZA has been observed to produce better effects than a lower dose [11], it is not clear whether in Asian patients low-dose (50–100 mg daily) AZA is as effective as the standard dose for long-term maintenance of remission. In China, where the incidence of CD is rapidly rising, the number of patients dependent on immunomodulators has increased over the years [12]. In the present study, we reviewed of all CD patients who underwent AZA therapy at our medical center and analyzed the efficacy and tolerance of AZA.

## Methods

### Patients population

The clinical data of CD patients from 1993 to December 2012 in Ren Ji Hospital, Shanghai Jiao Tong University School of Medicine, Shanghai Institute of Digestive Disease were reviewed. The diagnosis of CD was made based on clinical, endoscopic, histopathological, radiological findings and at least 6 months follow-up [13]. The patients who received AZA treatment and were followed for ≥ 2 years were screened. Patients were excluded if 1) their medical data or follow-up data were not complete; 2) they received anti-tumor necrosis factor-alpha monoclonal antibody therapy for inducing remission within three months before/after AZA therapy. The disease activity was assessed by the Harvey and Bradshow Index (HBI). Data regarding dosage, concomitant medications, body weight, HBI, duration of remission, efficacy and adverse events were collected from the medical record or follow-up.

### The decision for using AZA

The decisions of administrating AZA were: 1) frequent relapse; 2) gastrointestinal stricture/obstruction; 3) fistulizing CD; 4) prevention of postoperative recurrence; 5) steroid-sparing agent for steroid-dependent or steroid-refractory; 6) moderate to severe CD. Clinical relapse was defined as HBI >4 or need for re-introduce of steroids or occurrence of new complications. Frequent relapse was defined as ≥2 relapses within 1 year. Steroid-dependence was defined as relapse after the dosage of steroids tapered to 10 mg or relapse within three months after steroids sparing. Steroid-refractory was defined as no response towards full dose of steroids for four weeks.

### Treatment strategies

The patients, who were prescribed with AZA, were monitored for full blood count once a week and liver function tests every 2 weeks within 4 weeks at the start of therapy, and monthly thereafter. The efficacy was evaluated every three months.

### The definition of efficacy and toxicity

Remission was defined as HBI ≤4 or a decrease of at least 2 of HBI. Leucopenia was defined as white blood cell count less than 3 × 10^9^/l (normal range: 4 × 10^9^/l-10 × 10^9^/l); Thrombocytopenia was defined as platelet count less than lower border of normal range (<100 × 10^9^/l). Hepatic injury was defined as a liver function test above the normal range.

### Statistics

Doses were presented as mean ± SD. Remission rates were expressed as a proportion with 95% confidence interval (CI). The decisions for AZA treatment and withdrawn were compared by chi-square. Clinical characteristics between groups were analyzed by Student’s *t*-test, chi-square, or Mann–Whitney *U*-test. Probabilities of constant remission or off-steroid remission were calculated using the Kaplan-Meier statistical method and tested using Log-rank test. A 2-sided *P*-value <0.05 was considered statistically significance.

## Results

In all, the data of 99 CD patients who started AZA treatment before December 2010 were investigated. Twenty-two patients were excluded: 8 (8.1%) due to the lack of complete medical records or follow-up data and 14 (14.1%) due to concomitant anti-tumor necrosis factor-alpha monoclonal antibody therapy within 3 months of AZA therapy. Finally, 77 patients who met the inclusion criteria were included in our study: 54 were male and 23 female. The mean age at the start of AZA treatment was 32.2 years (range, 15–68 years). The mean duration from diagnosis to AZA treatment was 27.6 months (range, 0–240 months). Twenty-eight patients (36.4%) had undergone abdominal operations prior to AZA treatment. Age at diagnosis, disease behavior and location were categorized according to the Montreal classification [14]: 30 (39.0%), 10 (13.0%), 35 (45.5%), and 15 (19.5%) patients were classified as L1 (ileum), L2 (colon), L3 (ilecolon), and L4 (upper gastrointestinal tract) respectively; and 33 (42.9%), 40 (51.9%), 7 (9.1%), and 24 (31.2%) patients were classified as B1 (nonstricturing, nonpenetrating), B2 (stricturing), B3 (penetrating), and p (perianal disease) respectively. The decisions for using AZA were summarized (Table 1).

**Table 1:** Decisions for using azathioprine

**Decisions**	**All Patients with AZA**	**<1.0 mg/kg group**	**1.0-2.0 mg/kg group**	***P-*** **value**
	**(n = 77)**	**(n = 39)**	**(n = 38)**	
Gastrointestinal stricture/obstruction	37	19	18	0.854
Fistulizing CD	22	10	12	
Frequent relapse	15	8	7	
Moderate to severe CD	10	4	6	
Prevention of postoperative recurrence	6	2	4	
Steroid-sparing agent for steroid-dependent or steroid-refractory	6	4	2	

18 cases contained more than one decision.

*AZA* azathioprine, *CD* Crohn’s Disease.

### Dose of AZA

Seventy-four patients (96.1%) started treatment with 50 mg/d of AZA, two (2.6%) with 75 mg/d, and one (1.3%) with 25 mg/d. The doses of AZA were adjusted according to efficacy and tolerance (Table 2). Significantly higher doses were administered in patients who didn’t achieve remission compared to those who achieved remission at 12 months (1.4 ± 0.4 versus 0.9 ± 0.2 mg/kg; *P* = 0.009) and 24 months (1.2 ± 0.5 versus 1.0 ± 0.2 mg/kg; *P* = 0.005), but not at 6 months (1.0 ± 0.2 versus 1.0 ± 0.3 mg/kg; *P* = 0.828). In our cohort, almost half the patients were treated with <1.0 mg/kg AZA. According to their initial dose, we divided the patients into two groups: <1.0 mg/kg group and 1.0-2.0 mg/kg group.

**Table 2:** Doses (mg/kg) of azathioprine at different time points

	**Mean dose**	**Range dose**	**Patients with <1.0 mg/kg**	**Patients with 1.0-2.0 mg/kg**	**Patients with >2.0 mg/kg**
Initial	1.0 ± 0.2	0.6-1.6	39 (50.6%)	38 (49.4%)	0
6 Month	1.0 ± 0.3	0.4-1.7	29 (50.0%)	29 (50.0%)	0
12 Month	1.0 ± 0.3	0.4-1.9	24 (51.1%)	23 (48.9%)	0
24 Month	1.1 ± 0.3	0.4-2.1	17 (43.6%)	21 (53.9%)	1 (2.6%)

**Figure 1:**
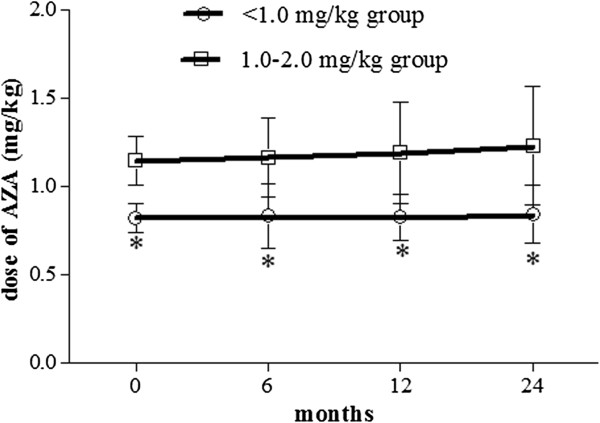
**Dose of azathioprine in <1.0 mg/kg group and 1.0-2.0 mg/kg group at different time points.** The patients treated with AZA were divided into <1.0 mg/kg group and 1.0-2.0 mg/kg group according to their initial dose. The doses of AZA were adjusted according to efficacy and tolerance during the 2-year period. The mean dose of <1.0 mg/kg group remained under 1.0 mg/kg. * *P* < 0.01 compared the dose of <1.0 mg/kg group with 1.0-2.0 mg/kg group.

Within 2 years, the doses of six patients in each group were adjusted: in <1.0 mg/kg group, four were increased and two decreased, and in 1.0-2.0 mg/kg group, five were increased and one decreased. The mean dose of <1.0 mg/kg group remained under 1.0 mg/kg during the 2 years, and a significant difference was found between the two groups (Figure 1).

### Efficacy of AZA treatment

**Figure 2:**
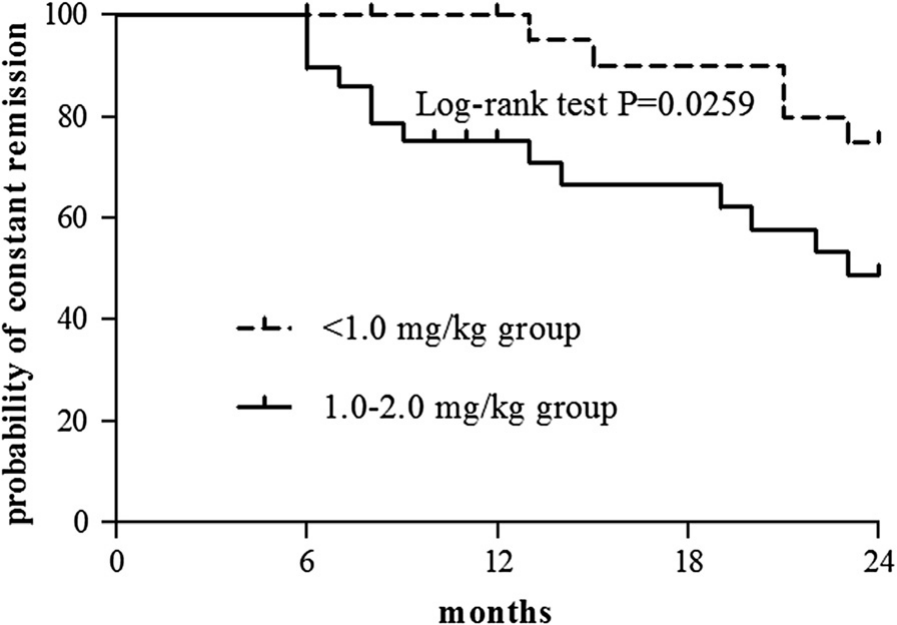
Cumulative probabilities of constant remission during 24 months using Kaplan-Meier statistical method.

The effect of AZA treatment was first evaluated at 6 months because remission was induced by corticosteroids in 62 patients (80.5%), and the doses of corticosteroids were continuously tapered during 3–6 months at the start of AZA treatment. The overall remission rates of all 77 patients at 0, 6, 12, and 24 months were 7.8% (95% CI, 3.2%-16.8%), 70.1% (95% CI, 58.5%-79.8%), 53.2% (95% CI, 41.6%-64.6%), and 35.1% (95% CI, 24.8%-46.9%) respectively. The percentage of patients who continued taking AZA at 6, 12, and 24 months were 75.3% (95% CI, 64.0%-84.1%), 61.0% (95% CI, 49.2%-71.7%), and 50.6% (95% CI, 39.1%-62.1%) respectively, and their remission rates were 93.1% (95% CI, 82.5%-97.8%), 87.2% (95% CI, 73.6%-94.7%), and 69.2% (95% CI, 52.3%-82.5%) respectively. When remission was achieved in relapsed patients after adjusting the treatment regimens, the remission rates were 93.1% (95% CI, 82.5%-97.8%), 93.6% (95% CI, 81.4%-98.3%), and 92.3% (95% CI, 78.0%-98.0%) respectively; the overall remission rates of 77 patients at 6, 12, and 24 months were 70.1% (95% CI, 58.5%-79.8%), 57.1% (95% CI, 45.4%-68.2%), and 46.8% (95% CI, 35.4%-58.4%) respectively. A significant difference was observed in the remission rates of the <1.0 mg/kg group and 1.0-2.0 mg/kg group (*P* = 0.0259) (Figure 2), and a significant difference was also observed in the off-steroid remission rates between two groups (*P* = 0.0120). The decisions for using AZA and the reasons for withdrawal of treatment were listed and no significant difference was found between the two groups (Tables 1 and 3).

**Table 3:** Causes for withdrawal of azathioprine treatment

**Causes**	**All Patients with AZA**	**<1.0 mg/kg group**	**1.0-2.0 mg/kg group**	***P-*** **value**
Adverse events	14	10	4	0.101
No response	3	0	3	
Relapse	1	1	0	
Patients’ intention	20	10^Δ^	10^Δ^	
Total	38	21	17	

^Δ^Four patients were aware of their white blood cells count <4 × 10^9^/l (and >3 × 10^9^/l), and discontinued AZA therapy by their own choices.

*AZA* azathioprine.

### Putative factors associated with low-dose AZA (<1.0 mg/kg)

In our cohort, there were more male patients in <1.0 mg/kg group than in 1.0-2.0 mg/kg group (*P* = 0.005). The mean body weight was significantly greater in <1.0 mg/kg group than in 1.0-2.0 mg/kg group (*P* < 0.001). In addition patients in <1.0 mg/kg group were older (35.5 years old) than those in 1.0-2.0 mg/kg group (28.7 years old) (*P* = 0.011), although there was no significant difference in age at diagnosis according to the Montreal classification. Locations significantly differed between the two groups (*P* < 0.001): L3 was more prevalent in 1.0-2.0 mg/kg group, while L1 was more prevalent in <1.0 mg/kg group (Table 4).

**Table 4:** Characteristics of patients between <1.0 mg/kg group and 1.0-2.0 mg/kg group

**Type**	**All Patients with AZA**	**<1.0 mg/kg group**	**1.0-2.0 mg/kg group**	***P-*** **value**
	**(n = 77)**	**(n = 39)**	**(n = 38)**	
Gender				
Male	54	33	21	0.005
Female	23	6	17	
Age at AZA treatment	32.2	35.5	28.7	0.011
Duration from diagnosis to AZA treatment	27.6	25.7	29.5	0.683
Age at diagnosis				
A1	5	2	3	0.112
A2	59	27	32	
A3	13	10	3	
Location				
L1	30	24	6	<0.001
L2	10	5	5	
L3	35	9	26	
L4	15	9*	6*	
Behavior				
B1	33	19	14	0.342
B2	40	19	21	
B3	7	2^#^	5^#^	
P	24	11	13	
Surgery history				
Yes	28	15	13	0.698
No	49	24	25	
Initial HBI	5.0	4.8	5.1	0.624
Initial body weight (kg)	52.9	60.5	45.2	<0.001
Concomitant with 5-ASA/SASP				
At initial				
Yes	50	29	21	0.079
No	27	10	17	
At month 24				
Yes	17	9	8	0.455
No	22	9	13	
Adverse events				
Yes	21	13	8	0.226
No	56	26	30	
Leucopenia				
Yes	8	4	4	1.000
No	69	35	34	

*Including L4 coexist with L1or L3.

^#^Including B3 coexist with B2.

*P* for difference between <1.0 mg/kg group and 1.0-2.0 mg/kg group.

*AZA* azathioprine, *HBI* Harvey and Bradshow Index, *5-ASA* 5-aminosalicylic acid, *SASP* sulfasalazine.

### Adverse events

Adverse events were observed in 21 of 77 patients (27.3%), due to which 14 (18.2%) discontinued AZA therapy (Table 5). Leucopenia occurred in 8 patients (10.4%), of whom three discontinued therapy. Infection occurred in 2 patients (2.6%) (One suffered from perianal fistula infected by *Staphylococcus* and AZA therapy was discontinued after recovery by the patient’s choice; another suffered from urinary tract infection). However, no significant difference was observed in the occurrence of adverse events or leucopenia between <1.0 mg/kg group and 1.0-2.0 mg/kg group (Table 4).

**Table 5:** Adverse events of azathioprine in 77 Patients

**Type**	**Adverse events**	**AZA withdrawal**	**Concomitant with 5-ASA/SASP**
Leucopenia	8	3	3
Hepatic Injury	7	5	4
Thrombocytopenia	1	1	1
Pancreatitis	1	1	1
Infection	2^φ^	1	1
Rash of Skin	1	1	1
Discomfort of Stomach	1	1	0
Fatigue	1	1	1
Total	22	14	12

^φ^Including both leucopenia and infection occurred in one patient.

*5-ASA* 5-aminosalicylic acid, *SASP* sulfasalazine.

## Discussion

In Europe and the United States, the dose of AZA for effective induction and maintenance of remission, as recommended by consensus, is 2.0-2.5 mg/kg [1,8]. A previous study has shown that for the majority of Koreans, low-dose AZA (1.35 mg/kg, 105 patients) for remission induction of inflammatory bowel disease was as effective as the standard dose (2.25 mg/kg, 17 patients), and 0.94-1.68 mg/kg was effective for CD maintenance. However, almost all patients in the low-dose group developed leucopenia or neutropenia, indicating that the doses were too strong for the patients [15]. In Japan, 0.6-1.2 mg/kg AZA was prescribed for patients with ulcerative colitis, but only 17 out of 22 (77.3%) patients completed that 6-month trial [16]. These reports suggest that in eastern Asia, the suitable dose of AZA is much lower than the standard, and that the effect of a <1.0 mg/kg dose of AZA for maintenance of remission is limited. In the present study on Chinese patients, the mean doses at 6, 12, and 24 months were 1.0, 1.0, and 1.1 mg/kg respectively, and almost half the patients were treated with <1.0 mg/kg AZA. The efficacy of ~1.0 mg/kg AZA in maintaining remission of CD was confirmed. Next, we compared the effect of <1.0 mg/kg AZA with that of 1.0-2.0 mg/kg AZA at 24 months, based on our previous result that the severity of disease remained unaltered in 90% patients with CD during the 2-year period [17]. To our surprise, the remission rate in patients of <1.0 mg/kg group was significantly higher than that in those of 1.0-2.0mg/kg group. To explain this unexpected result, we further analyzed the factors associated with <1.0 mg/kg group.

At our medical center, the doses of AZA were adjusted according to efficacy and tolerance, and the doses of patients who did not achieve remission were significantly higher than those of patients who achieved remission at 12 and 24 months. We do not know whether the need-based escalation of doses in <1.0 mg/kg group reached ≥1.0 mg/kg. The doses of six patients in each group were adjusted: in <1.0 mg/kg group, four were increased and two decreased, and in 1.0-2.0 mg/kg group, five were increased and one decreased. The mean AZA dose of <1.0 mg/kg group remained under 1.0 mg/kg during the 2-year period, and was lower than that of 1.0-2.0 mg/kg group. Since the remission rate increased after treatment regimens were adjusted, we were unable to determine whether the patients discontinued AZA treatment or whether the decisions for using AZA influenced dose adjustment. No significant differences were observed between the two groups with regard to the causes for withdrawal of AZA treatment and the decisions for using AZA. Adverse events were the main reason for AZA withdrawal and they hindered dose escalation. No significant difference was observed with respect to the occurrence of adverse events or leucopenia between the two groups. In vivo, when AZA was concomitantly administered with 5-aminosalicylic acid sulfasalazine (5-ASA/SASP), the level of 6-thioguanine nucleotide significantly increased with high occurrences of leucopenia [18]. We hypothesize that the concomitant administration of 5-ASA/SASP may be related to the lower dose AZA. In the present study, no significant difference was found with regard to the concomitant use of 5-ASA/SASP between the two groups at 0 and 24 months. Adverse events were not significantly affected by the concomitant administration of 5-ASA/SASP between the groups.

Our results indicated that a dose of <1.0 mg/kg AZA was more commonly associated with male gender, older age, heavier body weight, and L1 location. Male gender has been identified as a factor for long-term remission [19,20], and in the present study it was also related with low-dose AZA (<1.0 mg/kg). On the other hand, higher rates of relapse were seen in female patients; their doses of AZA had to be escalated. Older age was another factor for long-term remission and young patients were prone to be disabling CD [17,19-21]. Patients in <1.0 mg/kg group were significantly older than those in 1.0-2.0 mg/kg group at the start of AZA therapy, and the age (35.5 years) was almost the same as that reported by Fraser AG et al., who observed a lower relapse rate in the patients above 36 years of age [19]. Heavier body weight was also related with low-dose AZA (<1.0 mg/kg). In fact, the patients with heavier body weight suggested CD did not markedly influence the health of patients. In the previous reports, location had no significant effect on the treatment of AZA, except for colon, which was implicated to be associated with remission [19,20]. In our cohort, only 10 (13.0%) patients were classified as L2, L1 (39.0%) and L3 (45.5%) were the majority. Significant differences were seen with regard to the locations between two groups: L3 was more prevalent in 1.0-2.0 mg/kg group, while L1 was more prevalent in <1.0 mg/kg group. It is plausible that patients with larger extent of disease required higher dose of AZA to maintain remission. Overall, male gender, older age, heavier body weight, and L1 location were associated with long-term remission using <1.0 mg/kg AZA.

Leucopenia (white blood cells count <3 × 10^9^/l) has been reported to occur in 20-30% patients taking 2.0-2.5 mg/kg AZA [22]. In the present study, leucopenia occurred in 10.4% patients. In fact, a white blood cell count of <4 × 10^9^/l was seen in 24.7% patients. If the patients were aware of their low white blood cell counts (<4 × 10^9^/l and >3 × 10^9^/l), they would have discontinued AZA therapy by their own choices (eight patients in the present study). Since there was no difference in the relapse rates between leucopenic and non-leucopenic patients, we did not escalate the dose up to development of leucopenia [15]. Our principle of prescription was to adjust the dose of AZA according to the white blood cell count and HBI: if HBI was <4 and AZA was well tolerated, the same dose of AZA was continued. We believed that some patients in our cohort were capable of tolerating higher doses of AZA, but chose not to because their HBI was <4 and they were satisfied with the therapy. Our patients’ wishes were always respected. Thus, the principles of prescription practiced by our medical center also contributed to the lower dose AZA.

Our results showed that in approximately half the patients with CD, low-dose AZA (<1.0 mg/kg) proved efficient for the maintenance of remission and the principles of prescription applied by our medical center was suitable. In Europe and in the United States, the dose of AZA for CD patients is escalated every 2 weeks until the target dose of 2.0-2.5 mg/kg is reached [23]. Western and Eastern countries differ not only in the manifestations of CD but also with regards to its treatment [12,24]. The limitations of this study are its small sample size, retrospective nature and single-center design. A previous report found that 2.0 mg/kg AZA was well tolerated by 11 of 13 Chinese patients [25] and that Singapore Asians could tolerate 1.95 mg/kg AZA [26]. These results suggest that a prospective, multicenter, large clinical trial is necessary to confirm the efficacy of <1.0 mg/kg AZA for long-term maintenance of remission in Chinese patients with CD.

## Conclusions

In summary, in Chinese patients with CD, low-dose AZA (<1.0 mg/kg) may be effective for the maintenance of remission, and dose adjustment should be individualized according to efficacy and adverse events.

## Abbreviations

AZA: Azathiopurine; CD: Crohn’s disease; HBI: Harvey and Bradshow Index; SASP: Sulfasalazine; 5-ASA: 5-aminosalicylic acid.

## Competing interests

The authors declare that they have no competing interests.

## Authors’ contributions

JW was responsible for acquisition of data, analysis, and preparing the manuscript. YG and XY were assisted in acquisition of data and drafting the manuscript. XL and SX were involved in discussion and revised the manuscript. CY was responsible for design, analysis and interpretation of data. All authors have read and approved the final manuscript.
